# The effect of collagen hydrogels on chondrocyte behaviors through restricting the contraction of cell/hydrogel constructs

**DOI:** 10.1093/rb/rbab030

**Published:** 2021-07-01

**Authors:** Longpeng Dong, Qingli Liu, Yongli Gao, Hengxing Jia, Wenling Dai, Likun Guo, Hongsong Fan, Yujiang Fan, Xingdong Zhang

**Affiliations:** National Engineering Research Center for Biomaterials, Sichuan University, 29 Wangjiang Road, Chengdu, Sichuan 610064, PR China

**Keywords:** type I collagen, contraction, photo-crosslinkable hydrogel, chondrocyte

## Abstract

Collagen is a promising material for tissue engineering, but the poor mechanical properties of collagen hydrogels, which tend to cause contraction under the action of cellular activity, make its application challengeable. In this study, the amino group of type I collagen (Col I) was modified with methacrylic anhydride (MA) and the photo-crosslinkable methacrylate anhydride modified type I collagen (CM) with three different degrees of substitution (DS) was prepared. The physical properties of CM and Col I hydrogels were tested, including micromorphology, mechanical properties and degradation properties. The results showed that the storage modulus and degradation rate of hydrogels could be adjusted by changing the DS of CM. *In vitro*, chondrocytes were seeded into these four groups of hydrogels and subjected to fluorescein diacetate/propidium iodide (FDA/PI) staining, cell counting kit-8 (CCK-8) test, histological staining and cartilage-related gene expression analysis. *In vivo*, these hydrogels encapsulating chondrocytes were implanted subcutaneously into nude mice, then histological staining and sulfated glycosaminoglycan (sGAG)/DNA assays were performed. The results demonstrated that contraction of hydrogels affected behaviors of chondrocytes, and CM hydrogels with suitable DS could resist contraction of hydrogels and promote the secretion of cartilage-specific matrix *in vitro* and *in vivo*.

## Introduction

Tissue engineering is using ideal scaffolds to encapsulate cells for the purpose of new tissue regeneration, and it has the potential to achieve long-term satisfying results compared to traditional clinical repair methods such as marrow stimulation in cartilage repair [1–3]. Collagen is the primary component of chondrocyte extracellular matrix (ECM) and plays an important role in cartilage tissue engineering because of its excellent biocompatibility and its ability to support cell adhesion and proliferation [4]. The limited source and complicated extraction process of type II collagen have restricted its application. In contrast, Col I is widely used due to its wide source, low immunogenicity, and its ability to provide a cartilage-like microenvironment for cells [5]. The literature has reported that mesenchymal stem cells in Col I hydrogels can be induced to differentiate into chondrocytes in the absence of growth factors *in vitro* [6], reflecting the excellent properties of Col I.

However, since collagen hydrogels formed by non-covalent crosslinking, they will contract rapidly under the action of cellular activity [7]. Many studies have shown that the excessive contraction of hydrogels often leads to the dedifferentiation of chondrocytes, the limitation of cell proliferation, the massive staining of Col I matrix, and the contraction is often related to the formation of fibrocartilage [8, 9]. Some scholars have pointed out that the degree of hydrogels contraction is determined by cell concentration and mechanical properties of the scaffolds [10, 11].

In order to resist cell-mediated contraction and prevent the dedifferentiation of chondrocytes, it is necessary to introduce chemical crosslinking into collagen hydrogels to improve their mechanical property. As a natural biological cross-linker, genipin has been used to chemically crosslink cartilage-derived matrix, which prevents scaffold contraction and promotes stem cell differentiation into chondrocyte [9]. However, chemical crosslinking agents usually have potential biocompatibility issues due to their cytotoxic nature [12].

Photo-crosslinking is a relatively non-cytotoxic method to crosslink collagen [13]. In this study, methacrylamide groups were introduced into collagen to endow it with photo-chemical reactivity and photo-chemical crosslinking ability. Three kinds of CM hydrogels with different physical properties were prepared by adjusting the degrees of substitution (DS) of CM, and their ability in maintaining chondrocyte phenotype was further explored *in vitro* and *in vivo*.

## Materials and methods

### Materials

Col I was obtained from the skin of neonatal calves according to the methods reported by previous scholars [14]. Photoinitiator Irgacure2959, Methacrylic anhydride (MA), fluorescein diacetate (FDA) and propidium iodide (PI) were all acquired from Sigma-Aldrich (St. Louis, USA). Safranine O, hematoxylin and eosin (H&E) were purchased from Solarbio (Beijing, China). Acetic acid and sodium hydroxide were acquired from Kelong Chemical (Chengdu, China).

### Synthesis of methacrylate anhydride modified type I collagen

The methacrylate anhydride modified collagen was prepared according to the methods reported by previous scholars [13, 15]. In brief, the pH value of the 2 mg/ml collagen solution (0.5 M acetic acid) was adjusted to 8.0 with 5 M sodium hydroxide. A certain amount of methacrylate anhydride (MA) was poured into the solution and stirred continuously at 4°C for 4 h. At the end of reactions, the solution was moved to a dialysis bag with a molecular weight of 8000–14 000 Dalton. The dialysis bag was sequentially dialyzed in mixed solution of 0.5 M acetic acid and ethanol with the concentrations of 80%, 50% and 20% for 3 h, respectively. Then the products were dialyzed in 0.5 M acetic acid solution for 4 days. CM with 30%, 50% and 80% theoretical degree of substitutions was synthesized by adding different amounts of MA, which was named as CM30, CM50 and CM80, respectively.

### Characterization of methacrylate anhydride modified type I collagen

Five milligram of Col I, CM30, CM50 and CM80 were dissolved in 0.6 ml of DMSO respectively, then the nuclear magnetic resonance spectrometer (AV11-400MH, bruker) was used to detect the ^1^H NMR spectra of the samples. The DS was calculated according to the proton integral area of double bond and benzene ring using the previously reported method [16].

### Fabrication of hydrogels

One milliliter of 0.5 M acetic acid solution was added into the centrifuge tube containing 20 mg of Col I or CM and placed at 4°C to dissolve the sample completely. The photoinitiator solution was added to the solution, then the solution was regulated to neutral with NaOH. The final concentration of the samples was 10 mg/ml and the final concentration of the photoinitiator was 0.5 mg/ml. The precursor solution was filled into a cylindrical mold with 6 mm wide and 2 mm high. The precursor solution was placed at 37°C for 15 min and then exposed to UV light (OminiCure S1500, EXPO, Canada) for 30 s to prepare hydrogels. The wavelength of UV light was 320–480 nm, and the power density was 8 W/cm^2^.

### Characterization of hydrogel

#### Morphology of hydrogels

The microscopic morphology of hydrogels was viewed by scanning electron microscopy (SEM, Hitachi S-4800, Japan). The hydrogels were washed with deionized water for three times, then the hydrogels were frozen with liquid nitrogen and immediately put into the freeze dryer for freeze-drying. After being cut off, the cross-section of samples was covered with a gold/platinum layer and then viewed by SEM.

#### Mechanical analysis

The hydrogels were prepared and soaked in PBS to achieve swelling equilibrium, then the mechanical properties of hydrogels were characterized with a dynamic mechanical analyzer with frequencies of 1, 2 and 5 Hz at room temperature, and the amplitude was 20 µm, prestress was 1 mN. Three parallel samples in each group were detected, and the results were averaged.

#### Degradability measurement

The initial mass of hydrogels was weighed, which was recorded as W_1_. Then the hydrogels were immersed in 50 µg/ml collagenase I solution at 37°C. At fixed time point, the mass was weighed and recorded as W_2_. Three parallel samples were detected to get the average value. The degradation rate of hydrogels was determined by the following equation:

$$\text{Degradation}\;\text{rate}=(W_{2}-W_{1})/W_{1}\times 100\%$$

### *In vitro* culture of chondrocytes in hydrogels

#### Chondrocyte isolation and encapsulation

Chondrocytes were obtained from the cartilage of neonatal rabbits using formerly reported methods [17]. The experimental animals were acquired from ENSIWEIER Biotechnology. All animal experiments in this study were authorized by the Animal Care and Use Committee of Sichuan University (approve number: KS2020372). The chondrocytes were incubated in α-MEM medium with 10% serum, 100 µg/ml penicillin and streptomycin solution and 50 µg/ml vitamin C. The culture dishes were placed in an environment containing 95% air and 5% CO_2_ at 37°C. In this study, the second-generation chondrocytes were collected and mixed with neutral precursor solution of hydrogels, and the final cell concentration was 5 × 10^6^ cells/ml. The cell/hydrogel constructs were prepared as described above and cultured in 24-well plate.

#### Live/dead staining

The survival of chondrocytes was detected by FDA/PI staining. Specifically, the cell/hydrogel constructs were rinsed using PBS and stained with FDA/PI dye (2 µg/ml) for 2 min. The live chondrocytes were stained green with FDA and the dead chondrocytes were stained red with PI. The confocal laser scanning microscope (CLSM, Leica-TCS-SP5, Germany) was used for the observation of constructs.

#### Cell proliferation

CCK-8 test was performed for the detection of cell proliferation in hydrogels. Specifically, the cell/hydrogel constructs were submerged in 500 µl of fresh serum-free medium with 10% CCK-8, then placed at 37°C and cultured for 4 h. The absorbance of the reacted solution at 450 nm was measured by a Microplate Reader (Multiskan FC, USA). Three parallel samples in each group were detected, and the results were averaged.

#### Cytoskeleton staining

The cytoskeleton of chondrocytes was stained red by rhodamine-phalloidin and the nucleus was stained blue by DAPI. Specifically, the cell/hydrogel constructs were fixed in 4% paraformaldehyde solution for 15 min. Subsequently, the 0.1% Triton X-100 solution was used to rupture cell membrane for 5 min. Then the constructs were stained with rhodamine-phalloidin dye for 1 h at room temperature and stained with DAPI for 15 min at 37°C. All the staining processes were performed at dark. Lastly, the cytoskeleton was observed with CLSM.

#### Histological evaluation

After being embedded with paraffin, the cell/hydrogel constructs were sectioned by a microtome (RM2235, Leica, Germany), then stained histologically to analyze cell morphology and ECM secretion. The morphology of chondrocytes was observed with H&E staining. Cartilage-specific ECM can be stained purple with toluidine blue (TB) and orange with safranin O (SO).

#### Biochemical assay

The cell/hydrogel constructs were collected after 2 and 3 weeks of culture, then they were digested in papain buffer solution at 60°C for 12 h. After digestion, they were centrifuged at 4000 rpm/s for 3 min in a centrifuge, and the supernatant was collected for testing. The content of sulfated glycosaminoglycan (sGAG) was quantified by the Blyscan sGAG assay kit (Biocolor, Newtown abbey, UK), then the absorbance value of the solution at 656 nm was determined by a Microplate Reader, and the content of sGAG in the samples was calculated using a standard curve. The DNA content was quantified according to the standard procedure of the DNA Quantification Kit (Pico Green, Invitrogen). The fluorescence intensity of the sample solution was determined at an excitation wavelength of 480 nm by a Microplate Reader, and the DNA concentration was calculated using a DNA standard curve. Three parallel samples in each group were detected, and the results were averaged.

#### Real-time quantitative polymerase chain reaction analysis

After being extracted from cells with the RNeasy Mini Kit (Qiagen, Germany), the mRNA was reverse transcribed to cDNA using the iScript cDNA Synthesis Kit (Bio-Rad, USA), then the cDNA was quantified by quantitative polymerase chain reaction (qPCR) using the SsoFast EvaGreen Supermix (Bio-Rad, USA). In this study, the gene expression level of Col II, Aggrecan, Sox9, Col X and Col I was measured and normalized to GAPDH. The primer sequences of the relevant genes involved in this study were shown in Table 1.

**Table 1.** Primers for qPCR amplification

Target	Forward primer (5′-3′)	Reverse primer (5′-3′)
GAPDH	ATCACTGCCACCCAGAAGAC	GTGAGTTTCCCGTTCAGCTC
Col II	GCCACCGTGCCCAAGAAGAACT	ACAGCAGGCGCAGGAAGGTCAT
Agg	CCTACCAGGACAAGGTCTCG	ACACCTTTCACCACGACCTC
Sox9	GGAAGCTCTGGAGACTGCTG	CGTTCTTCACCGACTTCCTC
Col I	AGAGGACCACGTGGAGAAAG	CCATCAAACTGAGCAGCAAA
Col X	GGAAAACAAGGGGAGAGAGG	CCAGGAGCACCATATCCTGT

### *In vivo* culture of chondrocytes in hydrogels

#### Subcutaneous implantation in nude mice

Nude mice (∼20 g) were used for histological evaluation and biochemical assay *in vivo*. All nude mice that stayed one month in this study lived in the environment with a regular cycle (12 h of light and 12 h of dark) and were given water and food which could be freely accessed. The cell/hydrogel constructs were fabricated and implanted subcutaneously into the back of nude mice. At scheduled time points, nude mice were anesthetized with pentobarbital sodium salt solution by intraperitoneal injection and sacrificed, and the cell/hydrogel constructs were taken out from the back of nude mice for histological evaluation and biochemical assay.

#### Biochemical assay

The cell/hydrogel constructs were collected from the back of nude mice after cultured for 1, 2 and 4 weeks, and the content of sGAG and DNA was determined as described above. Three parallel samples in each group were detected, and the results were averaged.

#### Histological evaluation

The collected cell/hydrogel constructs were embedded in paraffin and sectioned. The sections were stained with H&E, TB and SO to analyze the morphology of chondrocytes and the secretion of ECM *in vivo*.

### Statistical analysis

The results were given as mean ± standard deviation (SD). The statistical significance between groups was analyzed with one-way analysis of variance and the significant difference levels were set at *P* < 0.05 (*), *P* < 0.01 (**), *P* < 0.001 (***).

## Results and discussion

### Synthesis and characterization of methacrylate anhydride modified type I collagen

**Figure 1.**
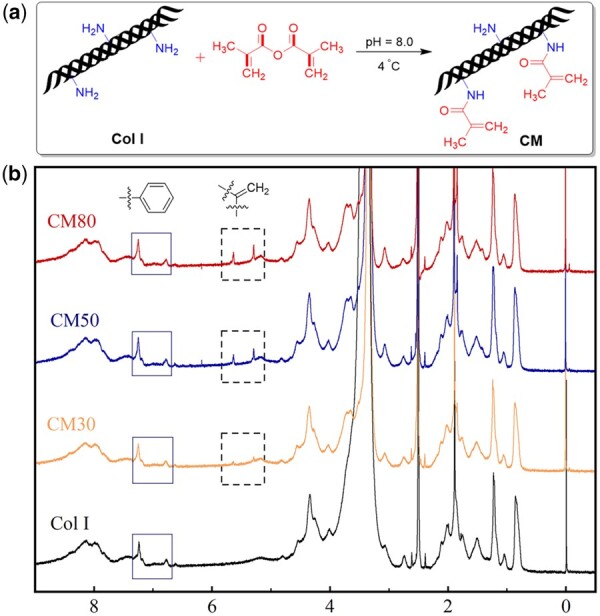
Synthetic scheme of CM (**a**) and ^1^H NMR spectra of Col I, CM30, CM50 and CM80 (**b**).

CM was prepared with Col I and MA at pH 8.0 and 4°C. The DS of CM was regulated by changing the proportion of Col I and MA. The synthetic scheme of CM was illustrated in Fig. 1a, and their chemical structures were confirmed by ^1^H NMR spectrum as shown in Fig. 1b. CM had absorption peaks at 5.3 and 5.6 ppm in ^1^H NMR spectrum, which were the proton peaks of the methacrylamide double bond (–C=CH_2_). The integral area ratio of absorption peaks of the methacrylamide double bond to benzene ring became larger as the DS of CM increased, indicating that the modification of collagen was successful and the CM with three different DSs was synthesized. The DS of CM was 27%, 48%, 76%, respectively, as calculated from ^1^H NMR spectra. These results confirmed that the DS of CM could be regulated by adjusting the ratio of Col I and MA.

### Fabrication and characterization of hydrogels

#### Fabrication of hydrogels

The Col I hydrogels formed by physical self-assemble through hydrogen bonding, electrostatic and hydrophobic interactions at 37°C [18], while CM hydrogels formed by both physical self-assemble and chemical reaction initiated by UV irradiation. In addition to self-assembling, the CM solution could form covalence through chemical reaction occurred by UV irradiation, so there was physical and chemical crosslinking occurrence based on self-assemble of CM and chemical reaction of methacrylamide groups in CM hydrogels.

#### Morphology observation

**Figure 2.**
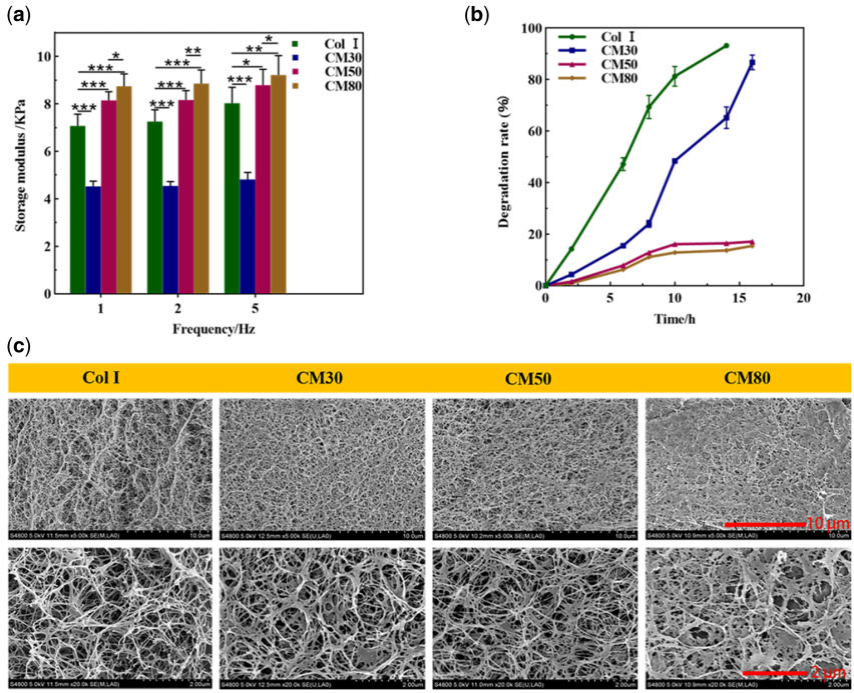
The storage modulus of hydrogels at different frequencies (1, 2 and 5 Hz) (**a**), the degradation curves of hydrogels measured in PBS containing 50 µg/ml collagenase I at 37°C (**b**) and SEM images of freeze-dried hydrogels (**c**) (*P < 0.05, **P < 0.01, ***P < 0.001).

The internal morphology of hydrogels has a significant impact on cell behaviors, nutrient delivery and metabolite excretion [19]. In this study, the microstructure of hydrogels was observed by SEM and the images were shown in Fig. 2c. The Col I hydrogels had continuous fibrous network structure and well interconnected pores, which could provide the encapsulated cells with a suitable microenvironment for cell proliferation and matrix secretion as reported [20]. The CM hydrogels had not only fibrous structure but also obvious lamellar structure, and they had more lamellar structure and became more compact as the DS of CM increased. It indicated that the morphology of hydrogels was closely related to its crosslinking manners and crosslinking density. Some studies have shown that chondrocytes grow faster and secrete more ECM in larger pores, but in smaller pores the cells often show a dedifferentiated form [21]. Microstructure of the scaffold materials such as pore size and pore volume has an effect on matrix secretion and growth of cells [22]. It has been reported that the proliferation ability and biological activity of the chondrocytes encapsulated in chitosan matrix will be improved as the interconnected pore size increases [23]. In this study, these findings suggested that the differences in the microstructure of hydrogels may have a critical impact on cellular activity.

#### Mechanical property of hydrogels

Many studies have shown that the stiffness of hydrogels has a significant effect on cell behaviors, such as cell proliferation, cell morphology, and the formation of new tissues [24–26]. In this study, the storage modulus of hydrogels was measured at the frequencies of 1, 2 and 5 Hz. The results (Fig. 2a) showed that the storage modulus of CM hydrogels increased with the increase of DS, and the storage modulus of CM50 and CM80 was 8.1 kPa and 8.7 kPa at the frequency of 1 Hz, respectively, while CM30 was only 4.5 kPa. The storage modulus of Col I was 7.1 kPa at the frequency of 1 Hz, which was significantly higher than CM30 but lower than CM50 and CM80. It indicated that the mechanical property of hydrogels was effectively regulated by crosslinking manners, meanwhile the crosslinking density of hydrogels also had a great impact on their mechanical properties. In these CM hydrogels, the higher DS meant the higher possibility of chemical crosslinking between collagen fibers, which was the reason that the storage modulus of CM hydrogels increased with increase of DS. Compared to Col I hydrogels, the presence of methacrylamide groups may partly hinder the physical self-assemble of CM30, meanwhile the density of chemical crosslinking was low owing to the lower DS of CM30, and this may be the reason that the mechanical property of CM30 was lower than that of Col I hydrogels. It can be speculated that the mechanical properties of hydrogels are closely related to crosslinking manners, and the formation of chemical bonds among molecules can usually give the materials higher mechanical strength.

#### In vitro degradation

The degradation property of hydrogels is a critical factor to consider in clinical applications [27]. The hydrogels can provide adhesion and proliferation sites for cells in tissue engineering, and their rate of degradation should match the generation of new tissue [28]. Collagen is a stable protein and its controlled degradation is critical during physiological development [29]. In this study, the hydrogels were soaked in collagenase I solution at 37°C and the weight loss percentage was measured at fixed time points as shown in Fig. 2b. It can be clearly seen that the degradation rate of hydrogels was closely associated with the DS of CM. Col I, in which hydrogels formed by physical self-assemble, had the fastest degradation rate. CM hydrogels formed by physical and chemical crosslinking, and the higher DS of CM led to the slower rate of degradation. It has been reported that the higher density of chemical crosslinking of hydrogels leads to lower degradation rate [30]. The degradation of collagen hydrogels was initiated by cleavage of the three chains between residues 775 and 776 by collagenase [29]. The structure of collagen hydrogels became more compact by chemical crosslinking and this steric hindrance effect prevented the diffusion of collagenase to the cleavage sites [31, 32]. Besides, previous reports have indicated that the presence of hydrophobic groups in hydrogels makes it hard for collagenase to reach the cleavage sites [33]. In this study, the introduction of hydrophobic methacrylamide groups can resist the attack of collagenase and limit its degradation ability. Based on above evidence, it can be speculated that the chemical crosslinking method contributes to the stable structure of hydrogels and slows down the rate of degradation.

### Chondrocytes culture *in vitro*

#### Cell survival and proliferation

**Figure 3.**
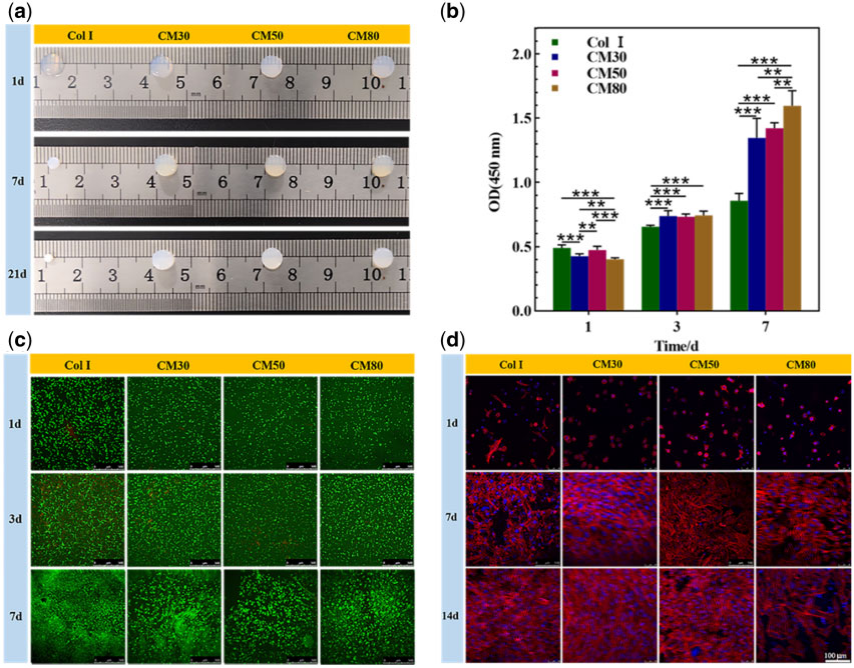
The appearance of cell/hydrogel constructs after being cultured for 1, 3 and 7 days *in vitro* (**a**), CCK-8 test for the proliferation of chondrocytes encapsulated in hydrogels (**b**), the images of live/dead staining (**c**) and cytoskeleton staining (**d**) (**P < 0.01, ***P < 0.001).

In order to evaluate the biocompatibility of hydrogels as tissue engineering scaffolds, live/dead staining was performed for observation of cell survival in hydrogels and CCK-8 test was performed for quantitative analysis of cell proliferation. The appearance of cell/hydrogel constructs after cultured for different intervals *in vitro* was shown in Fig. 3a. It can be seen that the Col I hydrogels contracted severely with the extension of culture time. However, the CM hydrogels did not contract obviously for up to 3 weeks of culture. It is reported that the movement of cells in the hydrogels may generate a large tension field and exert traction forces to hydrogels in order to push themselves outward, then causing significant contraction of the hydrogels [34]. It indicated that the chemical crosslinking helped to resist cell-mediated contraction of hydrogels in this study. The result of CCK-8 test demonstrated that the proliferation of chondrocytes in CM hydrogels was better than in Col I hydrogels as shown in Fig. 3b. Some studies have showed that cells ceased dividing when gels contracted, which may be attributed to the fact that the contraction of gels greatly reduced the space for cell growth and the cells stopped growing due to contact inhibition [35, 36]. The contracted hydrogels cannot provide an environment beneficial for the growth and proliferation of the encapsulated cells as reported [37]. The result of live/dead staining was shown in Fig. 3c, and it indicated that chondrocytes survived well in these hydrogels. The results suggested that these hydrogels were all biocompatible and the CM hydrogels might be more suitable for cell survival and proliferation compared to Col I hydrogels.

#### Cytoskeleton staining

Cell adhesion and spreading to the ECM is critical for tissue development and it directs cell survival, proliferation and the expression of differentiation phenotypes [38, 39]. The cytoskeleton staining result was shown in Fig. 3d and it demonstrated that all these hydrogels could promote chondrocytes adhesion, spreading and proliferation with the extension of culture time. Peptide sequences on collagen, including RGD and GFOGER, can provide adhesion sites for cells [40, 41]. It can be speculated that the modification of collagen in this study may not lead to a reduction of cell adhesion sites and has almost no bad effect on cell adhesion and spreading.

#### Biochemical assay and chondrogenic gene expression

**Figure 4.**
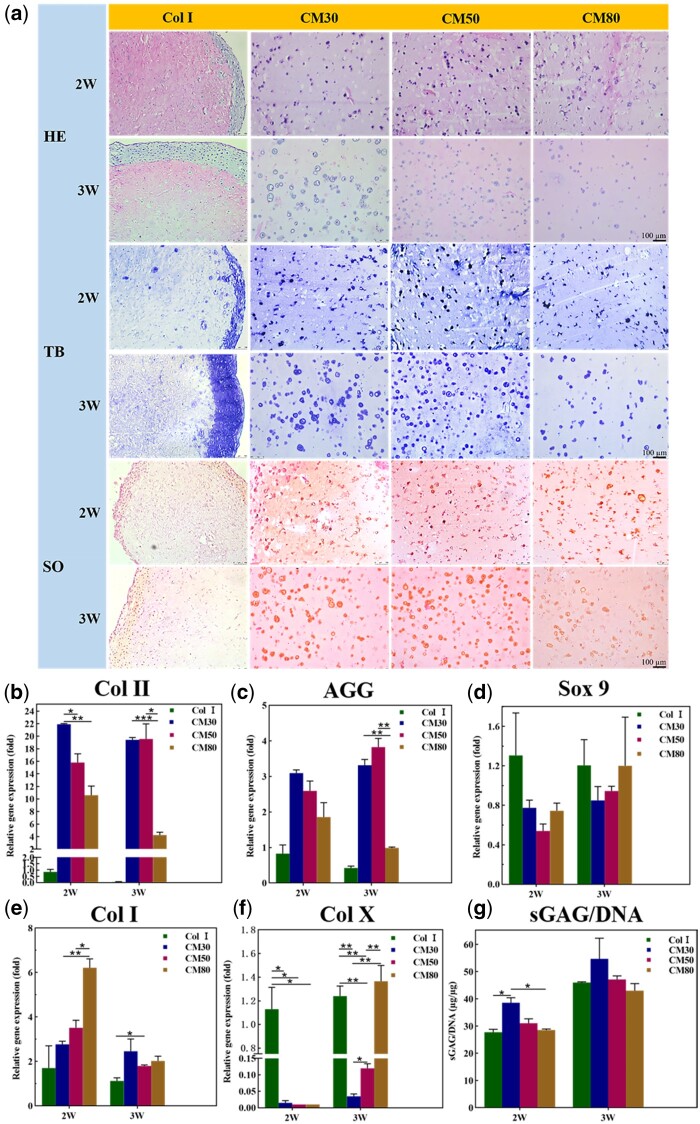
Histological staining (**a**), real-time qPCR quantification of gene expression associated with chondrogenesis (Col II (**b**), AGG (**c**) and Sox 9 (**d**)) or chondrocytes dedifferentiation (Col I (**e**), Col X (**f**)), and quantitative determination of sGAG/DNA (**g**) of cell/hydrogel constructs cultured *in vitro* for 2 and 3 weeks (*P < 0.05, **P < 0.01, ***P < 0.001).

The content of sGAG and DNA in cell/hydrogel constructs was measured to detect ECM secretion of chondrocytes in different hydrogels. The result showed that the value of sGAG/DNA in CM30 hydrogels was statistically higher than other groups as shown in Fig. 4g, which meant that chondrocytes in CM30 hydrogels had a greater ability to secrete glycosaminoglycans *in vitro*. The CM30 hydrogels could resist cell-mediated contraction though they were minimally modified, which allowed them to maintain a more porous microstructure that was beneficial for nutrient transport than CM50 and CM80. The severe contraction of Col I hydrogels may adversely affect the normal metabolic activity of chondrocytes as reported [35–37].

The gene expression level associated with chondrogenesis [type II collagen (Col II), aggrecan (AGG), and Sox9] and chondrocytes dedifferentiation [type I collagen (Col I), type X collagen (Col X)] was analyzed by real-time PCR and the results were shown in Fig. 4b–f. The results showed that cells in CM30 and CM50 hydrogels had higher gene expression level of key articular marker gene of Col II and AGG. Some studies have shown that cells synthesized little collagen in contracted hydrogels [42], so the lowest Col II and AGG gene expression level of cells in Col I hydrogels may be attributed to the severe contraction of hydrogels. The gene expression level of Sox 9 had no significant difference between all groups. The cells in CM80 hydrogels had the highest gene expression level of Col I at 2 weeks, and the expression level significantly decreased in all groups when the culture duration was extended to 3 weeks. It indicated that the chondrocytes in CM80 hydrogels may be more prone to dedifferentiation than in other groups due to the higher expression of Col I at the early culture duration. The gene expression level of Col X was significantly higher in cells encapsulated in Col I and CM80 hydrogels than that in CM30 and CM50 hydrogels at 3 weeks, and the value of *P* between CM30 and CM80 hydrogels was 0.0051. These findings indicated that chondrocytes were prone to hypertrophy in Col I and CM80 hydrogels, while CM30 and CM50 hydrogels might be beneficial for hyaline cartilage formation *in vitro*.

#### Histological staining

The histological staining was performed to observe chondrocyte morphology and cartilage-specific ECM secretion after cell/hydrogel constructs were cultured for 2 or 3 weeks. The chondrocytes in all hydrogels showed round morphology by H&E staining as shown in Fig. 4a, but more cartilage lacunas were observed in CM30 and CM50 hydrogels. Meanwhile, it can be found that lots of chondrocytes aggregated at the edge of Col I hydrogels. The possible reason for the concentration of chondrocytes at the edge of Col I hydrogels was that the hydrogel contraction led to densification of the collagen fibers, which limited the cell growth space and the supply of nutrients to the inside of the gel [43]. Secreted ECM was detected by TB staining and SO staining as shown in Fig. 4a, and the results demonstrated that there was more ECM secreted in CM30 and CM50 hydrogels. Similar to the results of H&E staining, the secreted matrix in Col I hydrogels was also detected to concentrate at the edge of the hydrogel. The internal structure of hydrogels can influence the matrix secretion of cells [19]. The CM80 hydrogels may be not convenient for nutrient transport due to the lamellar structure resulted from the higher crosslinking density compared with others, which caused the lower ECM synthesis. These results *in vitro* suggested that the CM30 and CM50 hydrogels were more suitable for the maintenance of the chondrocyte phenotype and there was more ECM secretion compared to Col I and CM80 hydrogels.

### Chondrocytes culture *in vivo*

#### Biochemical assay

**Figure 5.**
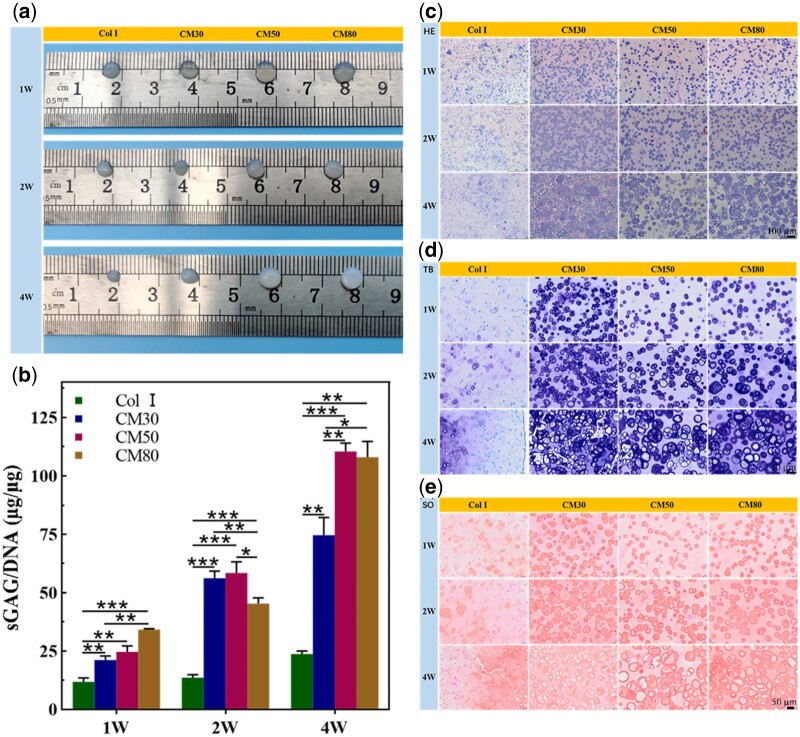
The appearance of cell/hydrogel constructs after a certain culture time *in vivo* (**a**), quantitative determination of sGAG/DNA (**b**), H&E staining (**c**), TB staining (**d**) and SO staining (**e**) of cell/hydrogel constructs after being cultured *in vivo* for 1, 2 and 4 weeks (*P < 0.05, **P < 0.01, ***P < 0.001).

Glycosaminoglycans are primary components of cartilage ECM [44]. The highly sulfated glycosaminoglycans are covalently bound to the core proteins of proteoglycans, which are crucial for cartilage mechanical property [45]. The appearance of hydrogels in Fig. 5a showed that Col I and CM30 hydrogels both contracted with the extension of culture time and the former was more severe. The contraction of CM30 may be attributed to the mechanical force of cells on the gels and the complex mechanical environment *in vivo*. Cells would reorganize collagen fibers to form a non-covalent stable structure resulting in the contraction of gels and smaller gaps between adjacent collagen fibers [46]. The contraction of hydrogels may lead to enhanced mechanical interaction between the cells and the scaffold materials.

The value of sGAG/DNA can reflect the cartilage-specific ECM secretory ability of chondrocytes. In this study, the content of sGAG and DNA in hydrogels was measured separately after being cultured for 1, 2 and 4 weeks *in vivo*. The result of sGAG/DNA in Fig. 5b indicated that the ECM secretion ability of chondrocytes in CM hydrogels was statistically higher than that in Col I hydrogels, and the *P* value between Col I and CM50 hydrogels was 0.00096 at 4 weeks. The value of sGAG/DNA in CM50 and CM80 hydrogels was significantly higher than that in CM30 hydrogels at 4 weeks, and the *P* value between CM30 and CM50 hydrogels was 0.0094. These results demonstrated that the hydrogel contraction *in vivo* limited the maintenance of the chondrocyte phenotype and discouraged chondrocytes from secreting-specific matrix.

#### Histological staining

The results of HE, TB and SO staining *in vivo* experiments were shown in Fig. 5. The H&E staining (Fig. 5c) showed that there were significant cartilage lacunas in CM hydrogels in the later stages compared to that in Col I hydrogels when the implantation time was extended to 4 weeks. It can be seen that there were a large number of cell clusters in the CM hydrogels, which may be caused by cell migration and proliferation. The TB and SO staining (Fig. 5d and e) indicated that chondrocytes in CM hydrogels secreted a large amount of ECM, and the ECM-specific staining was more obvious than that in Col I hydrogels. However, a small amount of cartilage lacunas and matrix secretion were observed in the Col I hydrogels. These results suggested that there was more ECM secretion in CM hydrogels compared to that in Col I hydrogels, and it is consistent with the result of sGAG/DNA. Previous studies have demonstrated that photo-crosslinked collagen hydrogels can promote mesenchymal stem cell differentiation toward chondrogenesis by inhibiting the expression of contraction-related signaling pathways *in vivo*, while the contraction of collagen hydrogels can cause dedifferentiation by exerting mechanical stimulation on the cells and activating these signaling pathways [47]. Mechanical effects of the environment on cells regulated cell adhesion, migration, gene expression and fate [48]. Chondrocytes respond to mechanical overload by disrupting the composition and structure of the ECM, but sustained mechanical overload can damage the normal function of chondrocytes [49]. In this study, the severe contraction of hydrogels may promote the hypertrophy trend of chondrocytes by reducing the ability to maintain their phenotype due to the excessive mechanical stimulation exerted on the chondrocytes. It can be speculated that a certain degree of modification of collagen to introduce chemically crosslinkable groups was beneficial for the maintenance of chondrocytes phenotype due to the limitation of hydrogel contraction.

## Conclusions

In this study, the modified collagen was prepared by controlling the ratio of MA to Col I. The CM hydrogels had stronger mechanical properties and slower degradation rate as DS of CM increased. CM hydrogels were able to resist cell-mediated contraction better than Col I hydrogels both *in vitro* and *in vivo*. CM30 and CM50 hydrogels promoted chondrocyte ECM secretion *in vitro*, while CM50 and CM80 had better ability to promote chondrocyte ECM secretion *in vivo*. In summary, the MA-modified collagen showed some excellent properties and could be an ideal candidate for tissue engineering scaffolds.

## Funding

This work was sponsored by the National Key Research and Development Program of China (2019YFA0110600), Science and Technology Support Program of Sichuan Province (2019YJ0161) and Guangxi Key Research and Development Plan (GuikeAB16450003).

*Conflict of interest statement*. None declared.
